# Can *Giardia* Infection Impair the Diagnostic Level of Fecal Calprotectin in Patients with Inflammatory Bowel Disease? A Case Report

**Published:** 2018

**Authors:** Sara MOHAMMAD ALI GOL, Hamed MIRJALALI, Hamid ASADZADEH AGHDAEI, Mohammad Reza ZALI

**Affiliations:** 1. Behbood Research Center for Gastroenterology and Liver Diseases, Shahid Beheshti University of Medical Sciences, Tehran, Iran; 2. Foodborne and Waterborne Diseases Research Center, Research Institute for Gastroenterology and Liver Diseases, Shahid Beheshti University of Medical Sciences, Tehran, Iran; 3. Basic and Molecular Epidemiology of Gastrointestinal Disorders Research Center, Research Institute for Gastroenterology and Liver Diseases, Shahid Beheshti University of Medical Sciences, Tehran, Iran; 4. Gastroenterology and Liver Diseases Research Center, Research Institute for Gastroenterology and Liver Diseases, Shahid Beheshti University of Medical Sciences, Tehran, Iran

**Keywords:** Inflammatory bowel diseases, *Giardia intestinalis*, Fecal calprotectin test

## Abstract

Inflammatory bowel disease (IBD) is attributed to complex conditions of gastrointestinal tract that is frequently reported all over the world. Fecal calprotectin evaluation is described as a primary tool to screen IBD patients. There are reports showing the confounding role of some microbial agents in diagnostic levels of calprotectin. A 32-yr-old woman with symptoms like IBD/IBS (irritable bowel syndrome); admitted to IBD Clinic of Behbood Research Center for Gastroenterology and Liver Diseases, Shahid Beheshti University of Medical Sciences, Tehran, Iran, Jan 2017 for evaluation of the level of fecal calprotectin. In spite of high level of calprotectin, trophozoite of *Giardia intestinalis* was observed in direct examination of stool sample. Microbial pathogens can lead to false elevation of fecal calprotectin and misdiagnosis of gastrointestinal patients suspected to IBD.

## Introduction

*Giardia* spp., is a flagellated protozoan causing chronic diarrhea over the world. *G. intestinalis* (Syn. *G. duodenalis, or G. lamblia*) is known as one of the most prevalent causative agents of traveler’s diarrhea and waterborne/foodborne outbreaks. Currently, Giardiasis considered as neglected disease, but about 280 million people are annually infected with this parasite. Although infected patients with *G. intestinalis* are often asymptomatic, severe complications such as diarrhea, steatorrhea, nonspecific gastrointestinal symptoms, flatulence, excessive fatigue, nausea, foul-smelling stools, and weight loss are sporadically reported (1, 2). There are studies on prevalence of *Giardia* infection and molecular distribution of this parasitic protozoan in Iran that suggest a potential linkage between certain genotypes and diarrhea (3, 4).

IBD is a gastrointestinal disorder frequently reported in Iran (5, 6). Many biomarkers are introduced to screen patients suspected to IBD. Calprotectin is a protein that mainly released from leukocytes and epithelial cells during inflammation. This protein is suggested as a marker for primary screening and follows up of IBD patients. However, fecal calprotectin has 95% sensitivity and 91% specificity to monitor IBD patients (7–10).

Elevated level of fecal calprotectin proposed to be attributed to some other disorders/diseases such as acute or chronic gastrointestinal microbial infections (viral, bacterial or parasite), allergic colitis, celiac disease, Nonsteroidal anti-inflammatory drugs (NSAIDs)- induced enteropathy and colorectal cancer (11–16). Until now, some intestinal parasitic diseases have been reported that elevated concentration level of fecal calprotectin (17, 18). Therefore, elevated level of fecal calprotectin resulting from gastrointestinal pathogens should be considered as a confounding factor of primary screening of IBD patients.

The current study reports a young female with sudden and acute abdominal complaints with heavy giardiasis who had high level of calprotectin and was referred to the gastrointestinal clinic as IBD-suspected patient.

## Case report

A 32-yr-old female patient who had severe bloating, nausea, fatigue and abdominal pain for a week admitted to IBD Clinic of Behbood Research Center for Gastroenterology and Liver Diseases, Jan 2017. She traveled to abroad approximately 1 month before clinical symptoms and took prolonged antibiotic therapy for acute sinusitis. She had no previous history of any abdominal complaints. The blood tests revealed normal Complete Blood Count (CBC) consisted of White Blood Cell (W.B.C): 6600 /micL, Red Blood Cell (R.B.C): 4.7 mil/micL, Hemoglobin (Hb): 13.8 g/dl. Differential count of WBC was Neutrophils: 70%, Lymphocytes: 26%, Monocytes: 3% and eosinophils: 1%. Liver function tests such as SGPT, SGOT and Alkaline phosphatase were 44 U/L, 28 U/L, and 170 U/L, respectively. Celiac disease tests including IgA, EMA, tTg (IgA) were 180 mg/dL, Negative and 1.0 U/ml, respectively. IgA, EMA, and tTg (IgA) were analyzed by turbidimetry, Immunofluorescence (EUROIMMUN, Germany) and ELISA (ORIENTEC, Germany), respectively. Antigen of *Helicobacter pylori* was not detected in stool using rapid chromatographic immunoassay test. A fresh stool sample was taken and examined for intestinal parasites. Stool sample was loose, few greasy and yellow colored in the appearance.

**Fig. 1:**
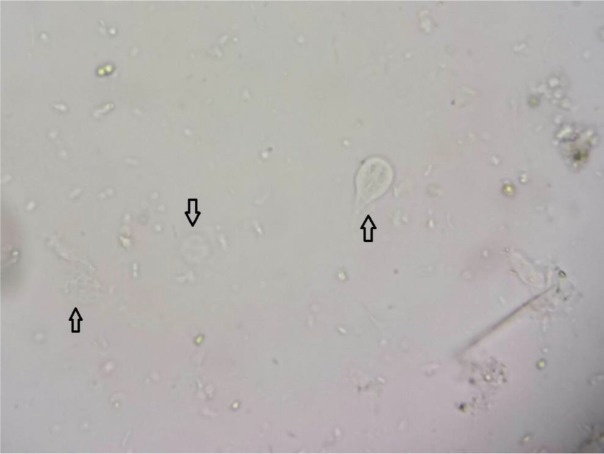
Trophozoites of *Giardia* sp., in fresh stool sample of an IBD patient characterized with arrows (400× magnification)

Trophozoite of *G. intestinalis* was detected in direct examination (Fig. 1). RBC and PMNs were not seen in microscopical examination of stool sample. It was not seen cyst of *G. intestinalis*, and other eukaryotic parasites and also ova of helminths in formalin-ethyl acetate concentration.

Primary fecal calprotectin was determined using an ELISA-based kit (EUROIMMUN, Germany) and revealed a high elevation level of calprotectin (2047 mg/kg).

The patient was treated with metronidazole (250 mg/kg), omeprazole (20 mg) and bismuth (120 mg), three times a day for 10 days. After treatment period, stool sample was taken and secondary fecal calprotectin determination was performed that showed a significantly decreased level. The second stool examination showed that there were no trophozoite and cyst of *G. intestinalis*.

After treatment, the general condition was well and there were no clinical complaints.

## Discussion

*G. intestinalis* is an intestinal protozoan that infects human subjects, particularly children. Fecal-oral transmission is known as primary route of infection, but food and drinking water are suggested as important sources of infection. *G. intestinalis* is one of the most important causes of traveler’s diseases frequently reported in Iran (4, 19, 20).

IBD is called to a complex of gastrointestinal disorders reported from Iran (21, 22). However, although some gastrointestinal diseases can lead to false elevation of fecal calprotectin level, fecal calprotectin is a serological test introduced for screening and differentiation between IBD and IBS patients (23). There are evidences showing the important role of microbial pathogens in elevation of the level calprotectin. It was seen correlation between severity of viral and bacterial infections and increased level of fecal calprotectin (13). The role of some parasitic diseases has also been discussed in false-increasing the diagnostic levels of fecal calprotectin in IBD patients. *G. duodenalis* can increase the level of fecal calprotectin (24). Furthermore, *Schistosoma mansoni* (intestinal schistosomiasis) was able to increase the level of fecal calprotectin (17). Heavy infection with some parasitic protozoan such as *Dientamoeba fragilis* leads to infiltrate neutrophiles in intestine (25) that can elevate the level of fecal calprotectin associated with inflammatory process (18).

## Conclusion

The current case supports the interesting role of some parasitic diseases in elevation the diagnostic levels of fecal calprotectin. Therefore, it is important to consider the probability of microbial infection in patients suspected to IBD who referred to gastrointestinal clinic.
